# Inhibition of IFN-γ-dependent antiviral airway epithelial defense by cigarette smoke

**DOI:** 10.1186/1465-9921-11-64

**Published:** 2010-05-26

**Authors:** Modestos A Modestou, Lori J Manzel, Sherif El-Mahdy, Dwight C Look

**Affiliations:** 1 Department of Internal Medicine, University of Iowa Carver College of Medicine, 200 Hawkins Drive, C33-GH, Iowa City, Iowa 52242-1081, USA

## Abstract

**Background:**

Although individuals exposed to cigarette smoke are more susceptible to respiratory infection, the effects of cigarette smoke on lung defense are incompletely understood. Because airway epithelial cell responses to type II interferon (IFN) are critical in regulation of defense against many respiratory viral infections, we hypothesized that cigarette smoke has inhibitory effects on IFN-γ-dependent antiviral mechanisms in epithelial cells in the airway.

**Methods:**

Primary human tracheobronchial epithelial cells were first treated with cigarette smoke extract (CSE) followed by exposure to both CSE and IFN-γ. Epithelial cell cytotoxicity and IFN-γ-induced signaling, gene expression, and antiviral effects against respiratory syncytial virus (RSV) were tested without and with CSE exposure.

**Results:**

CSE inhibited IFN-γ-dependent gene expression in airway epithelial cells, and these effects were not due to cell loss or cytotoxicity. CSE markedly inhibited IFN-γ-induced Stat1 phosphorylation, indicating that CSE altered type II interferon signal transduction and providing a mechanism for CSE effects. A period of CSE exposure combined with an interval of epithelial cell exposure to both CSE and IFN-γ was required to inhibit IFN-γ-induced cell signaling. CSE also decreased the inhibitory effect of IFN-γ on RSV mRNA and protein expression, confirming effects on viral infection. CSE effects on IFN-γ-induced Stat1 activation, antiviral protein expression, and inhibition of RSV infection were decreased by glutathione augmentation of epithelial cells using N-acetylcysteine or glutathione monoethyl ester, providing one strategy to alter cigarette smoke effects.

**Conclusions:**

The results indicate that CSE inhibits the antiviral effects of IFN-γ, thereby presenting one explanation for increased susceptibility to respiratory viral infection in individuals exposed to cigarette smoke.

## Background

A growing body of evidence indicates that cigarette smoke exposure increases susceptibility to viral (and bacterial) respiratory infection. It is well established that infants and children exposed to environmental tobacco smoke have an increased incidence and/or severity of otitis media and lower respiratory illness including respiratory syncytial virus (RSV) bronchiolitis compared to those not exposed [1-3]. Women passively exposed to cigarette smoke are at increased risk for more frequent common colds of longer duration [4]. Similarly, individuals that actively smoke cigarettes have an increased incidence and longer duration of respiratory infection [4,5]. Clinical upper respiratory infection is more common in smokers after controlled exposure to respiratory viruses [6]. It has also been reported that healthy army recruits that smoke cigarettes had a higher attack rate and more severe infection during an H1N1 influenza epidemic [7]. Thus, available information indicates that cigarette smoke increases the incidence, duration, and/or severity of respiratory viral infection. However, mechanisms responsible for the effects of cigarette smoke on lung defense are incompletely understood.

A central feature of the host response to viral infection in the airway is activation of epithelial cell genes that are important in innate and adaptive immunity by a potent group of mediators called interferons. Type II interferon (IFN) or IFN-γ is produced mainly by T cells and natural killer cells, and mediates host cell effects by binding to a specific receptor complex linked to a Janus kinase-signal transducer and activator of transcription (JAK-STAT) signaling cascade [8,9]. Activation of the type II interferon-driven pathway is triggered by engagement and multimerization of the IFN-γ-receptor (IFNGR) by IFN-γ, phosphorylation of IFNGR1-associated Jak1 and IFNGR2-associated Jak2 tyrosine kinases, and then phosphorylation of IFNGR1 [10]. Phosphorylation of the IFNGR1 chain of the IFN-γ receptor results in recruitment, phosphorylation, and subsequent release of Stat1 from the receptor [11]. Activated Stat1 dimerizes, translocates to the nucleus, and binds IFN-γ-activated sequence (GAS) elements in IFN-γ-inducible genes where it works in concert with adjacent transcription factors (e.g., Sp1) and coactivators (e.g., CBP/p300) to increase gene transcription [12,13]. IFN-γ-induced genes promote antiviral mechanisms that include leukocyte recruitment, antigen processing and presentation, cell proliferation and apoptosis, and antiviral state establishment.

Based on this information, we questioned whether cigarette smoke has direct effects on IFN-γ-dependent antiviral mechanism in airway epithelial cells that would impair host defense. In this report, we use primary human airway epithelial cells and an extract of cigarette smoke to demonstrate that this extract decreases antiviral effects of IFN-γ. These effects of cigarette smoke extract (CSE) are controlled, at least in part, through inhibition of Stat1 activation in epithelial cells. Furthermore, CSE effects on IFN-γ-dependent Stat1 activation and subsequent antiviral responses can be decreased by glutathione augmentation of epithelial cells suggesting that oxidants in cigarette smoke mediate a portion of these effects. Our results support the concept that exposure of the human airway to cigarette smoke directly impairs antiviral defense, thereby providing one explanation for increased respiratory virus susceptibility in individuals exposed to cigarette smoke.

## Methods

### Airway Epithelial Cell Isolation, Culture, and Treatments

Human trachea and bronchial samples from individuals without lung disease were obtained through the University of Iowa Cell Culture Core Repository under a protocol approved by the University of Iowa Institutional Review Board. Airways were dissected from lung tissue, and primary human tracheobronchial epithelial (hTBE) cells from the surface of airway mucosa were isolated by enzymatic dissociation. Cells were cultured in Laboratory of Human Carcinogenesis (LHC)-8e medium on plates coated with collagen/albumin as described previously [14,15]. To assure reproducible and generalizable results, experiments were repeated at least 3 times using hTBE cells from different individuals. The 12 individuals that provided epithelial cells were 29-76 years of age and included current smokers, ex-smokers, and nonsmokers. Some samples were treated with 100 units/ml of recombinant human IFN-γ (a gift from Genentech, South San Francisco, CA). In some experiments, hTBE cells were treated with the antioxidants N-acetylcysteine (NAC) or glutathione monoethyl ester (GSH-MEE) from Sigma-Aldrich (St. Louis, MO), at concentration of 5 mM and 1 mM, respectively. Time course schematics are included above each experiment figure to clarify the order and duration of cell treatments.

### Cigarette Smoke Extract

CSE was prepared by drawing mainstream smoke from the base of a lighted research reference cigarette (University of Kentucky) into a 60 ml syringe containing 10 ml of culture media. Smoke was drawn into the syringe 7 times with syringe capping and 100 shakes after each draw, resulting in combustion of the full length of the cigarette except for 0.5 cm adjacent to the filter. Consistency of the 100% CSE preparation was monitored by spectrophotometric measurement of absorbance, resulting in A_300nm _= 2.52-2.94 that correlated with an added cigarette smoke nonvolatile mass of 0.48-1.20 mg/ml. CSE was used immediately after generation, and was diluted to 5 or 10% in culture media (pH 7.24-7.35, normal media pH 7.25-7.30) prior to exposure of hTBE cells.

### Enzyme-Linked Immunoassay

ICAM-1 levels on the surface of cell monolayers was determined using an enzyme-linked immunoassay as described previously [15-18].

### Immunoblot Analysis

Whole cell and nuclear protein extract preparation and immunoblot analyses were performed as described previously [14,19-22]. Primary antibodies used to detect specific cellular and nuclear proteins were: mouse IgG1 mAb clone 6 against human interferon regulatory factor-9 (IRF-9) from BD Transduction Laboratories (Lexington, KY); rabbit polyclonal IgG 4915 against human ICAM-1, rabbit polyclonal IgG 9172 against total human Stat1, and rabbit polyclonal IgG 9171 against tyrosine-701 phosphorylated human Stat1 from Cell Signaling Technology (Beverly, MA); rabbit polyclonal antiserum against human heat shock protein (HSP)-90 from Assay Designs (Ann Arbor, MI); mouse IgG2a mAb clone AC-74 against human β-actin from Sigma-Aldrich (St. Louis, MO); rabbit polyclonal IgG ab4742 against serine-727 phosphorylated human Stat1 from Abcam (Cambridge, MA); goat polyclonal IgG against human RSV proteins from Biodesign International (Saco, ME). Primary antibody binding was detected using donkey antigoat, goat antimouse, or goat antirabbit IgG conjugated to horseradish peroxidase (Santa Cruz Biotechnology, Santa Cruz, CA) and an enhanced chemiluminescence detection system (Amersham Biosciences, Uppsala, Sweden). Reprobing of membranes was done after washing twice in Restore™ buffer (Pierce, Rockford, IL) for 15 minutes at 37°C. In most experiments, radiographic film images were analyzed using ImageJ software [23]. To generate an integrated density level, band area was multiplied by the band mean gray value, and the integrated density for the protein of interest was divided by the corresponding β-actin level creating a ratio for each sample.

### Epithelial Cytotoxicity Assays

Mitochondrial activity was assessed by quantification of 3-(4,5-dimethylthiazol-2-yl)-5-(3-carboxymethoxyphenyl)-2-(4-sulfophenyl)-2H-tetrazolium (MTS) reduction to a colored formazan product in the presence of phenazine ethosulfate using a commercial kit (Promega, Madison, WI). Determination of plasma membrane permeability to ethidium homodimers in dead cells and intracellular esterase activity in live cells was performed using a commercial fluorescence-based viability and cytotoxicity kit (Molecular Probes, Eugene, OR) as described previously [19,20]. A positive control for 100% cell death was generated by adding the permeabilizing agent 1% saponin to cells.

### Viral Preparation

High concentration human RSV strain A2 was from Advanced Biotechnologies (Columbia, MD), where it was produced from HEp-2 cell lysates. RSV was incubated with epithelial cells for 24 hours in culture media at 37°C in 5% CO_2 _at a multiplicity of infection (MOI) of 0.01 or 0.5 based on plaque titration assay using Vero cells as described previously [19,20]. An MOI of 0.01 was used in experiments requiring viral mRNA detection and 0.5 was used in experiments detecting viral proteins. MOI levels were chosen that resulted in infection of only a small percentage of epithelial cells but gave easily detected viral molecule levels in order to allow quantification of an increase or decrease in infection.

### Realtime Reverse Transcription PCR mRNA Analysis

Total cellular RNA was isolated using a commercial spin column isolation kit (Stratagene, La Jolla, CA), and equivalent amounts (0.5-1 μg) were reverse transcribed using a commercial kit (Ambion, Austin, TX). Equal aliquots of the resulting cDNA were subjected to PCR using an iCycler iQ Fluorescence Thermocycler (Bio-Rad Laboratories, Hercules, CA) with SYBR Green I DNA dye (Molecular Probes, Eugene, OR), iTaq™ DNA Polymerase (Bio-Rad Laboratories), and the following primers designed with software by S. Rozen and H. Skaletsky http://www.genome.wi.mit.edu/genome_software/other/primer3.html: 1) RSV N-gene sense 5'-GCTCTTAGCAAAGTCAAGTTGAATGA-3' and antisense 5'-TGCTCCGTTGGATGGTGTATT-3'; and 2) human hypoxanthine phosphoribosyltransferase (HPRT) sense 5'-GCAGACTTTGCTTTCCTTGG-3' and antisense 5'-AAGCAGATGGCCACAGAACT-3' or sense 5'-TTGGAAAGGGTGTTTATTCTTC-3' and antisense 5'-TCCCCTGTTGACTGGTCATT-3'. PCR conditions included denaturation at 95°C for 3 minutes, and then 45 cycles of 94°C for 30 s, 60°C for 30 s, and 72°C for 30 s, followed by melting curve analysis. Fluorescence data was captured during annealing reactions, and specificity of the amplification was confirmed using melting curve analysis. Data was collected and recorded by iCycler iQ software (Bio-Rad Laboratories) and initially determined as a function of threshold cycle (C_t_). C_t _was defined as the cycle at which the fluorescence intensity in a given reaction tube rose above background, which was calculated as 10 times the mean standard deviation of fluorescence in all wells over the baseline cycles. Levels of mRNA are expressed relative to control HPRT levels, and calculated as 2^ΔCt^.

### Glutathione Assay

Epithelial cell reduced glutathione levels were determined using a commercial luminescence-based assay utilizing a reaction catalyzed by a glutathione S-transferase (Promega), and results were normalized to sample protein levels.

### Statistical Analysis

Enzyme-linked immunoassays, cytotoxicity and glutathione assays, realtime reverse transcription PCR mRNA analyses, and densitometry analyses were repeated multiple times to assure reproducible results and were analyzed for statistical significance using analysis of variance (ANOVA) for a factorial experimental design. The multicomparison significance level for the ANOVA was 0.05. If significance was achieved by one-way analysis, post-ANOVA comparison of means was performed using Bonferroni's or Tukey's tests [24].

## Results

### Cigarette Smoke Extract Decreases Type II Interferon-Dependent Gene Expression

**Figure 1:**
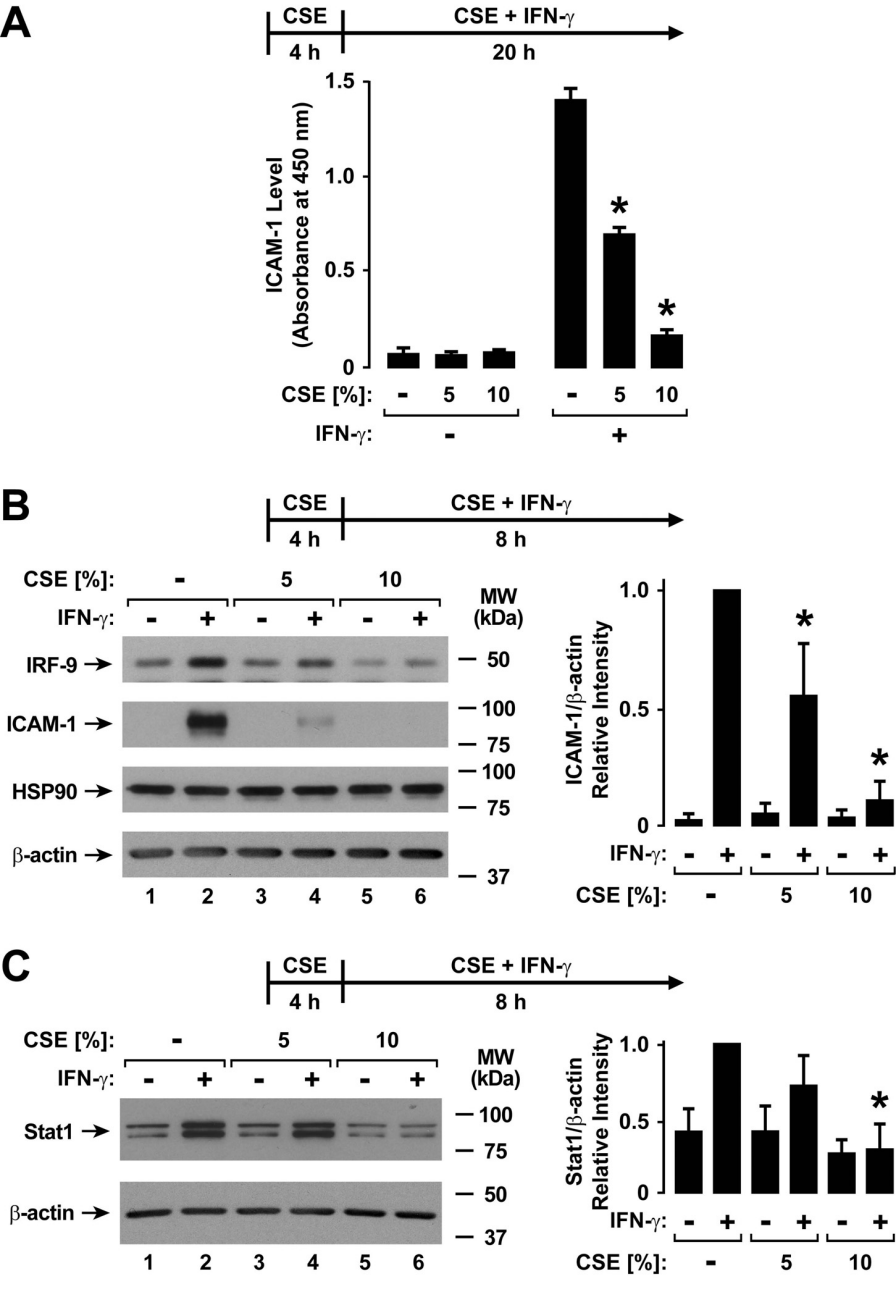
**Cigarette smoke extract decreases type II interferon-induced protein expression**. *A*: ICAM-1 cell surface protein levels were determined using an enzyme-linked immunoassay with hTBE cell monolayers that were first treated with media without or with CSE at the indicated concentration for 4 hours. Cells were then incubated for 20 hours in media containing the same CSE concentration without or with IFN-γ. Values are expressed as mean ± S.D. (*n *= 3 replicates), and a significant difference (*p *< 0.05) in IFN-γ-induced levels between cells treated versus not treated with CSE is indicated by an *asterisk*. *B*: IRF-9, ICAM-1, HSP90, and β-actin protein levels were assessed using immunoblot analysis of extracts from hTBE cells that were first treated with media without or with CSE at the indicated concentration for 4 hours, followed by incubation for 8 hours in media containing the same CSE concentration without or with IFN-γ. *C*: Total Stat1 and β-actin protein levels were assessed using immunoblot analysis of extracts from hTBE cells that were first treated with media without or with CSE at the indicated concentration for 4 hours, followed by incubation for 8 hours in media containing the same CSE concentration without or with IFN-γ. In *B *and *C*, protein levels were quantified using band densitometry of immunoblot analyses from separate experiments with the value for cells not exposed to CSE but treated with IFN-γ set at 1 in each experiment. Values are expressed as mean ± S.D. (*n *= 4 experiments) in the bar graph adjacent to a representative immunoblot analysis, and a significant difference (*p *< 0.05) in IFN-γ-induced levels between cells treated versus not treated with CSE is indicated by an *asterisk*.

Human airway epithelial cells are an important target for respiratory viral infection, and epithelial responses to interferons are critical for antiviral defense. The effects of type II interferon are primarily mediated through expression of cell proteins that regulate multiple antiviral defense mechanisms. To assess cigarette smoke effects on airway epithelial responses to IFN-γ, an extract of cigarette smoke was generated in culture medium and applied to hTBE cell monolayers prior to and during IFN-γ treatment and before viral infection. In this system, pretreatment of hTBE cells with CSE for 4 hours followed by addition of CSE during IFN-γ treatment inhibited the induction of the adhesive protein for leukocytes ICAM-1 seen with IFN-γ treatment for 20 hours (Figure 1A) or 8 hours (Figure 1B). IRF-9 (Figure 1B) and Stat1 (Figure 1C) are signaling proteins in which increased levels are also induced by IFN-γ, and this response was similarly decreased in cells treated with CSE. IRF-9, also known as interferon-stimulated transcription factor 3γ, is a component of the multiprotein transactivation complex that controls type I interferon-induced gene expression [25,26]. The form of Stat1 that was measured in these experiments is primarily the unphosphorylated form, and whether an increased level of this "substrate" for conversion to the active form of Stat1 affects IFN-γ-dependent signaling is unclear. The results suggest that CSE globally inhibits IFN-γ-induced protein expression in human airway epithelial cells.

### Cigarette Smoke Extract Causes Minimal Airway Epithelial Cell Cytotoxicity

**Figure 2:**
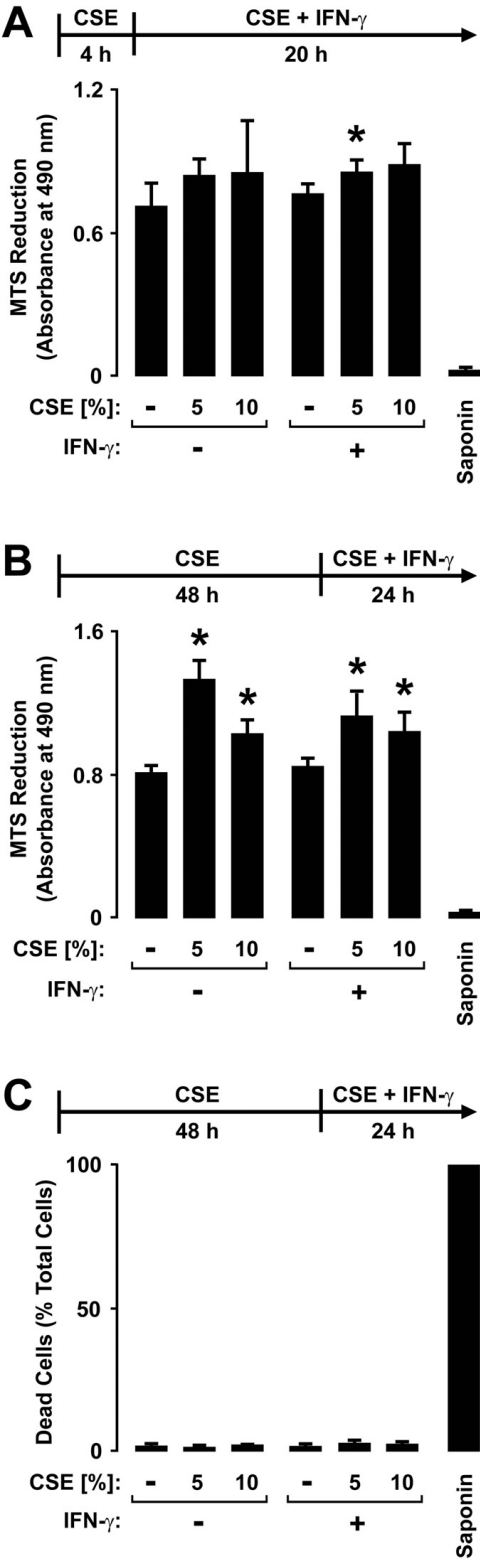
**Cigarette smoke extract causes minimal airway epithelial cell cytotoxicity**. *A*: Mitochondrial activity was quantified using an MTS-based assay with hTBE cells that were first treated with media without or with CSE at the indicated concentration for 4 hours, followed by incubation for 20 hours in media containing the same CSE concentration without or with IFN-γ. *B*: Mitochondrial activity was quantified using an MTS-based assay with hTBE cells that were first treated with media without or with CSE at the indicated concentration for 48 hours, followed by incubation for 24 hours in media containing the same CSE concentration without or with IFN-γ. In *A *and *B*, values are expressed as mean ± S.D. (*n *= 3 replicates). *C*: Dead and live hTBE cell numbers were quantified by detection of plasma membrane permeability to ethidium homodimers in dead cells and intracellular esterase activity in live cells. Cell monolayers were first treated with media without or with CSE at the indicated concentration for 48 hours, followed by incubation for 24 hours in media containing the same CSE concentration without or with IFN-γ. Values were calculated as dead cells/total cells and each condition represents the mean ± S.D. for 4 random low power fields (500-750 cells/field) from duplicate samples. In *A*-*C*, a significant difference (*p *< 0.05) in uninduced or IFN-γ-induced levels between cells treated versus not treated with CSE is indicated by an *asterisk*.

The effects of epithelial cell exposure to CSE on defense gene expression did not appear to be secondary to cell loss or cytotoxicity based on multiple parameters and assays of these effects. For example, no clear decrease in epithelial cell numbers or total cellular protein and RNA levels was detected in samples treated with CSE for 4-72 hours (M. A. Modestou, L. J. Manzel, D. C. Look, unpublished observations). Epithelial cells treated with CSE for 4 hours followed by incubation with CSE without or with IFN-γ for 20 hours resulted in only small changes in mitochondrial metabolic activity assessed using an assay of mitochondrial reductases in which MTS is converted to a formazan product (Figure 2A). Furthermore, extending the CSE pretreatment period to 48 hours resulted in increased MTT conversion, likely due to increased mitochondrial reductase activity rather than increased cell number (Figure 2B). Lastly, the combination of CSE for 4 hours (L. J. Manzel, D. C. Look, unpublished observation) or 48 hours (Figure 2C) followed by CSE without or with IFN-γ treatment did not significantly increase cell death as detected by plasma membrane permeability to ethidium homodimers in dead cells and intracellular esterase activity in live cells. Mean total epithelial cell numbers between conditions in the assay had less than 10% variability. Based on these results, we conclude that CSE effects on epithelial cell responses to IFN-γ are not due to cell death or cytotoxicity.

### Cigarette Smoke Extract Inhibits Type II interferon-Induced Stat1 Activation

**Figure 3:**
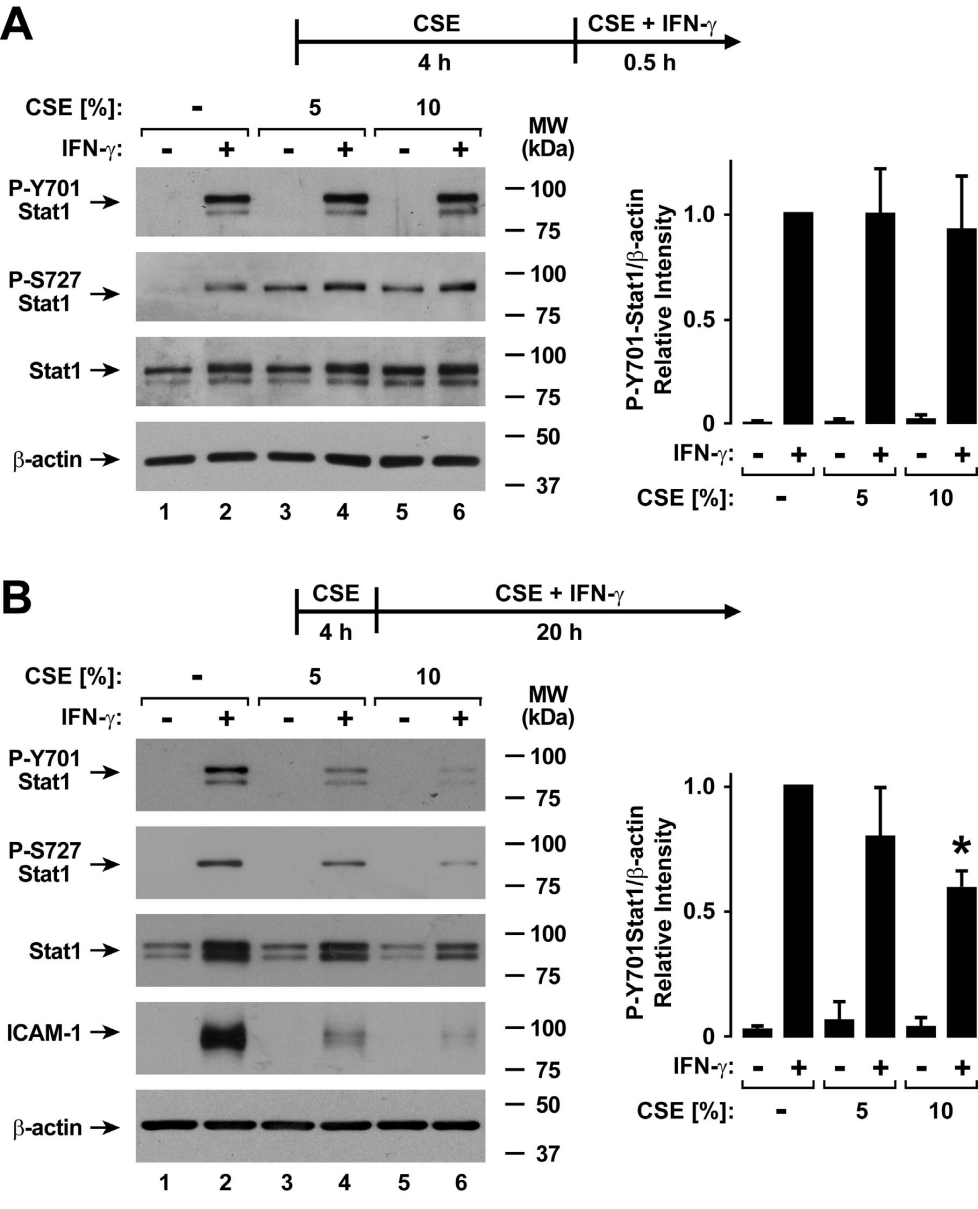
**Cigarette smoke extract inhibits type II interferon-induced Stat1 activation**. *A*: Tyrosine-701 and serine-727 phosphorylated and total Stat1, and β-actin levels were assessed using immunoblot analysis of extracts from hTBE cells that were first treated with media without or with CSE at the indicated concentrations for 4 hours, followed by incubation for 30 minutes in media containing the same CSE concentration without or with IFN-γ. *B*: Tyrosine-701 and serine-727 phosphorylated and total Stat1, ICAM-1, and β-actin levels were assessed using immunoblot analysis of extracts from hTBE cells that were first treated with media without or with CSE at the indicated concentrations for 4 hours, followed by incubation for 20 hours in media containing the same CSE concentration without or with IFN-γ. In *A *and *B*, protein levels were quantified using band densitometry of immunoblot analyses from separate experiments with the value for cells not exposed to CSE but treated with IFN-γ set at 1 in each experiment. Values are expressed as mean ± S.D. (*n *= 3 experiments) in the bar graph adjacent to a representative immunoblot analysis, and a significant difference (*p *< 0.05) in IFN-γ-induced levels between cells treated versus not treated with CSE is indicated by an *asterisk*.

The antiviral effects of type II interferon in epithelial and other cells requires activation of the transcription factor Stat1 by phosphorylation of tyrosine-701 and under some circumstances serine-727, with subsequent nuclear translocation and binding to gamma interferon activation sites in IFN-γ-responsive genes [27,28]. Based on initial results suggesting that CSE globally inhibits IFN-γ-dependent effects in human airway epithelial cells, we questioned whether CSE may affect Stat1 activation thereby providing a mechanism for CSE effects on type II interferon-mediated gene expression. Decreased Stat1 tyrosine-701 and serine-727 phosphorylation was not observed after 4 hours of CSE exposure followed by CSE and IFN-γ treatment for 30 minutes (Figure 3A). CSE alone induced Stat1 serine-727 phosphorylation after 4.5 hours of exposure independent of tyrosine-701 phosphorylation or IFN-γ treatment, but this had no effect on antiviral gene expression (Figure 1) and did not explain CSE effects on IFN-γ-induced gene expression. The observation that Stat1 phosphorylated on serine-727, but not tyrosine-701, did not affect gene expression correlated with findings that indicate tyrosine-701 phosphorylation is absolutely required for Stat1 transactivation function while serine-727 phosphorylation may under some conditions only augment this function [29]. In contrast to results with shorter CSE exposure, decreased Stat1 tyrosine-701 and serine-727 phosphorylation was seen when the duration of the combination of CSE and IFN-γ was extended to 20 hours (Figure 3B). Inhibition of IFN-γ-induced total (primarily unphosphorylated) Stat1 expression by CSE was also observed after 20 hours of IFN-γ treatment similar to results shown in Figure 1C. This effect is likely a consequence of the inhibition of Stat1 phosphorylation on the capacity for IFN-γ to induce Stat1's own gene transcription.

**Figure 4:**
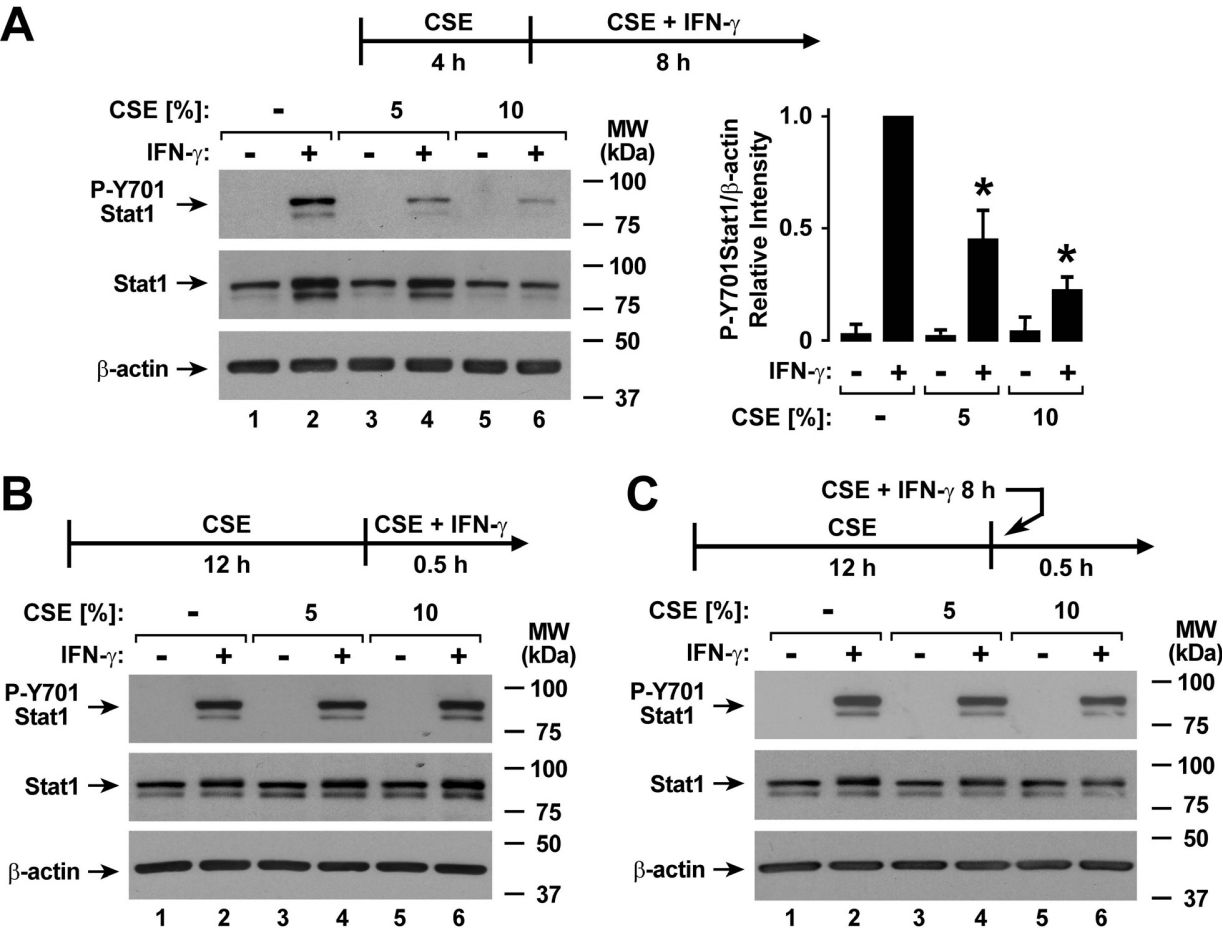
**Cigarette smoke extract has a delayed effect on epithelial cell Stat1 activation**. *A*: Tyrosine-701 phosphorylated and total Stat1, and β-actin levels were assessed using immunoblot analysis of extracts from hTBE cells that were first treated with media without or with CSE at the indicated concentrations for 4 hours, followed by incubation for 8 hours in media containing the same CSE concentration without or with IFN-γ. Protein levels were quantified using band densitometry of immunoblot analyses from separate experiments with the value for cells not exposed to CSE but treated with IFN-γ set at 1 in each experiment. Values are expressed as mean ± S.D. (*n *= 3 experiments) in the bar graph adjacent to a representative immunoblot analysis, and a significant difference (*p *< 0.05) in IFN-γ-induced levels between cells treated versus not treated with CSE is indicated by an *asterisk*. *B*: Tyrosine-701 phosphorylated and total Stat1, and β-actin levels were assessed using immunoblot analysis of extracts from hTBE cells that were first treated with media without or with CSE at the indicated concentrations for 12 hours, followed by incubation for 30 minutes in media containing the same CSE concentration without or with IFN-γ. *C*: Tyrosine-701 phosphorylated and total Stat1, and β-actin levels were assessed using immunoblot analysis of extracts from hTBE cells that were first treated with media without or with CSE at the indicated concentrations for 12 hours, followed by incubation for 30 minutes in media without or with IFN-γ that had been preincubated without or with the same CSE concentration alone for 8 hours.

Experiments in which the duration of CSE and IFN-γ treatment was varied revealed that inhibition of Stat1 activation occurred after 4 hours of CSE exposure followed by CSE and IFN-γ treatment for 8 hours (Figure 4A), but was not seen with 12 hours of CSE exposure followed by CSE and IFN-γ treatment for 30 minutes (Figure 4B). One possible explanation for this delayed effect could have been direct and time-dependent CSE inactivation of IFN-γ itself, but no clear loss of Stat1 activation was observed in hTBE cells if IFN-γ was preincubated with CSE alone in a tube without epithelial cells for 8 hours and then transferred to hTBE cells exposed to CSE alone for 12 hours (Figure 4C). The findings indicate that CSE effects on IFN-γ-induced cell signaling require a period of epithelial cell exposure to both CSE and IFN-γ.

### Cigarette Smoke Extract Decreases Type II Interferon-Dependent Antiviral Defense

**Figure 5:**
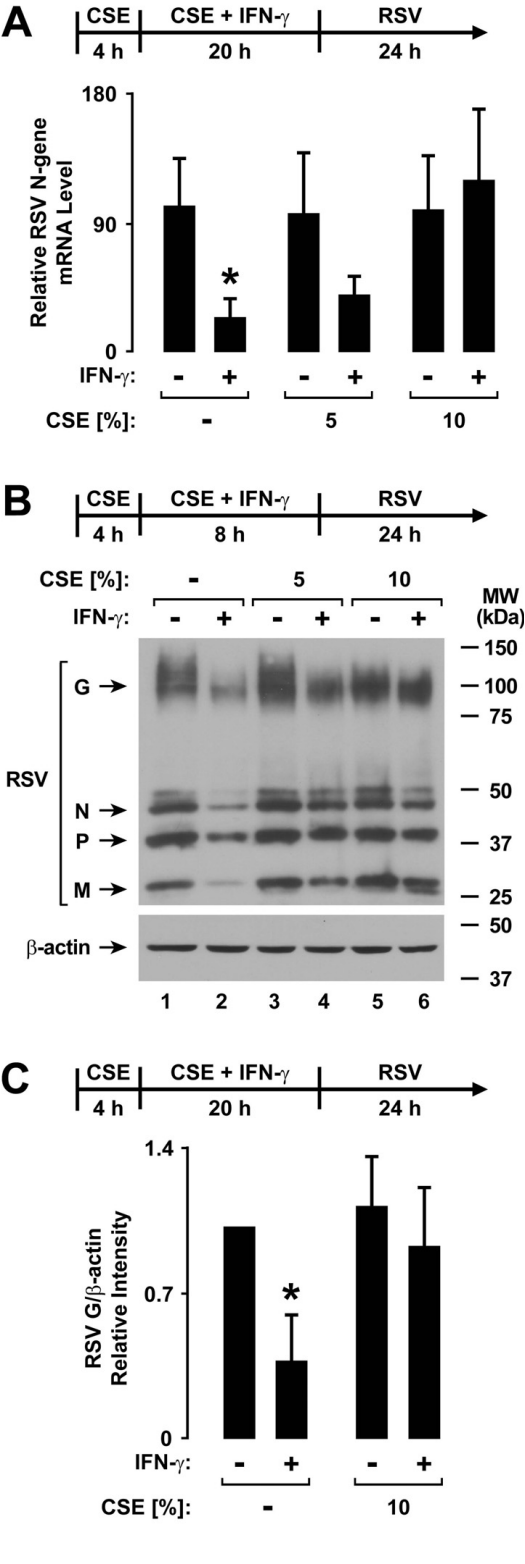
**Cigarette smoke extract decreases type II interferon-dependent antiviral defense**. *A*: RSV N-gene mRNA levels were determined using realtime RT-PCR analysis of total RNA from hTBE cells that were first treated with media without or with CSE at the indicated concentration for 4 hours, followed by incubation for 20 hours in media containing the same CSE concentration without or with IFN-γ. Cells were then infected with RSV for 24 hours. Values are expressed as mean normalized N-gene mRNA level compared to control HPRT mRNA level (*n *= 3-4 experiments), and a significant difference (*p *< 0.05) in untreated or CSE-treated levels between cells uninduced versus induced by IFN-γ is indicated by an *asterisk*. *B*: RSV and β-actin protein levels were assessed using immunoblot analysis of extracts from hTBE cells that were first treated with media without or with CSE at the indicated concentration for 4 hours, followed by incubation for 8 hours in media containing the same CSE concentration without or with IFN-γ. Cells were then infected with RSV for 24 hours. *C*: Protein levels were quantified using band densitometry of immunoblot analyses from separate experiments performed as outlined for *B*. The band intensity of RSV G/β-actin was set at 1 in each experiment for cells not exposed to CSE or IFN-γ and other conditions were compared relative to this value. Values are expressed as mean ± S.D. (*n *= 4 experiments), and a significant difference (*p *< 0.05) in untreated or CSE-treated levels between cells uninduced versus induced by IFN-γ is indicated by an *asterisk*.

Treatment of epithelial cells with IFN-γ prior to RSV infection significantly decreased viral N-gene mRNA expression assessed by realtime RT-PCR analysis (Figure 5A). Since RSV mRNA expression directly correlates with viral replication in epithelial cells, these results confirm the antiviral effects of type II interferon [30]. CSE inhibited the interferon-dependent decrease in viral mRNA expression, resulting in no significant difference in RSV N-gene mRNA expression without or with IFN-γ treatment. As viral protein expression correlates with viral mRNA levels and replication, we went on to assess the level of multiple viral proteins using immunoblot analysis. Similar to findings of CSE effects on viral mRNA levels, treatment of epithelial cells with IFN-γ prior to RSV infection decreased the levels of multiple RSV proteins in hTBE cells, but exposure to CSE inhibited IFN-γ effects that decreased RSV protein expression (Figure 5B and 5C). The results indicate that CSE inhibits IFN-γ-induced antiviral effects against RSV in human airway epithelial cells, and this correlates with effects on type II interferon-dependent signaling and gene expression.

### Glutathione Augmentation Inhibits Cigarette Smoke Extract Effects on Antiviral Defense

**Figure 6:**
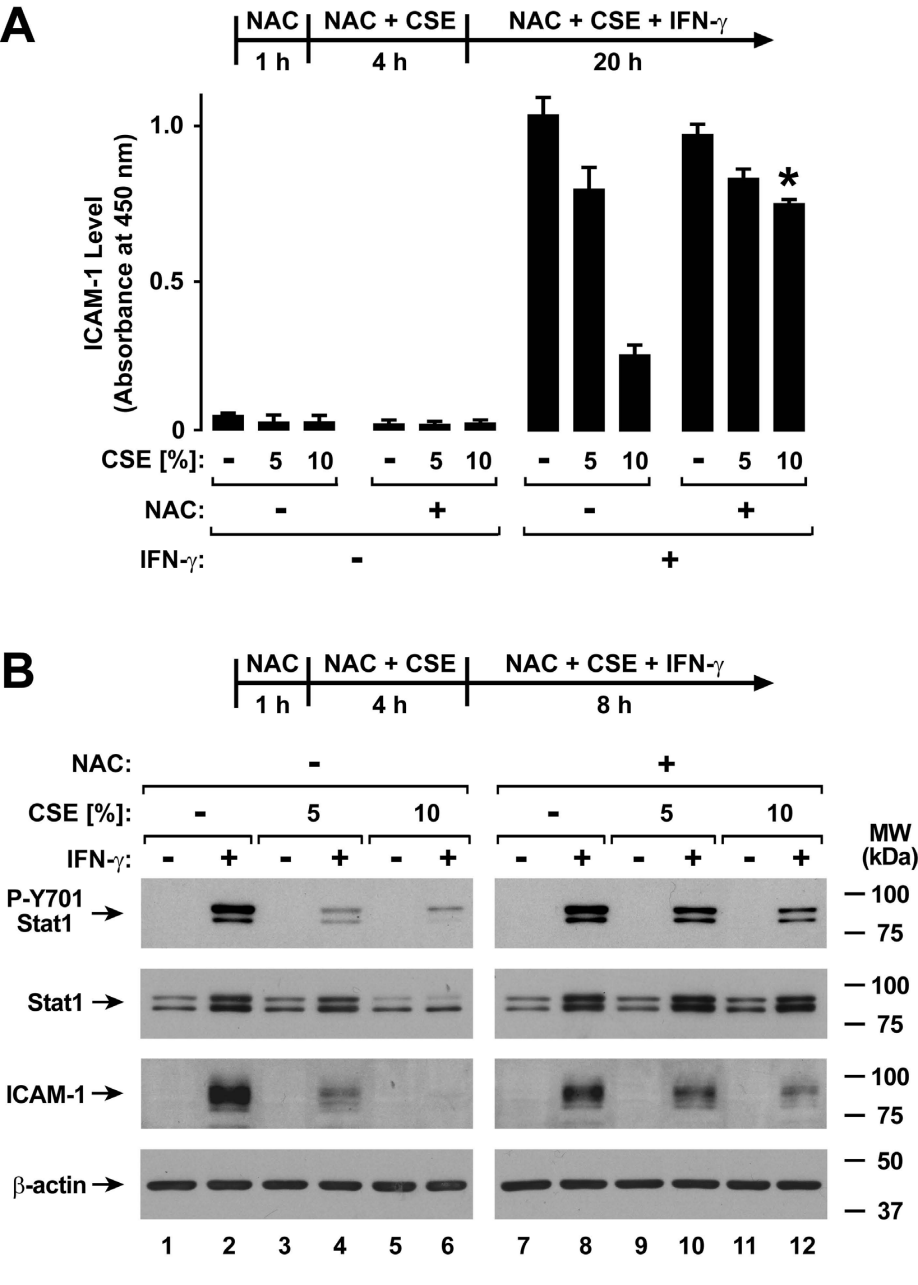
**N-acetylcysteine inhibits cigarette smoke effects on type II interferon-induced responses**. *A*: ICAM-1 cell surface protein levels were determined using an enzyme-linked immunoassay with hTBE cells that were first treated in media without or with NAC for 1 hour. Cells were then incubated in media containing the antioxidant without or with CSE at the indicated concentrations for 4 hours, followed by incubation for 20 hours with the same compounds without or with IFN-γ. Values are expressed as mean ± S.D. (*n *= 3 replicates) and a significant difference (*p *< 0.05) in comparable CSE-treated levels between cells not incubated versus incubated with NAC is indicated by an *asterisk*. *B*: Tyrosine-701 phosphorylated and total Stat1, ICAM-1, and β-actin protein levels were assessed using immunoblot analysis of extracts from hTBE cells that were first treated in media without or with NAC for 1 hour. Cells were then incubated in media containing the antioxidant without or with CSE at the indicated concentrations for 4 hours, followed by incubation for 8 hours with the same compounds without or with IFN-γ.

**Figure 7:**
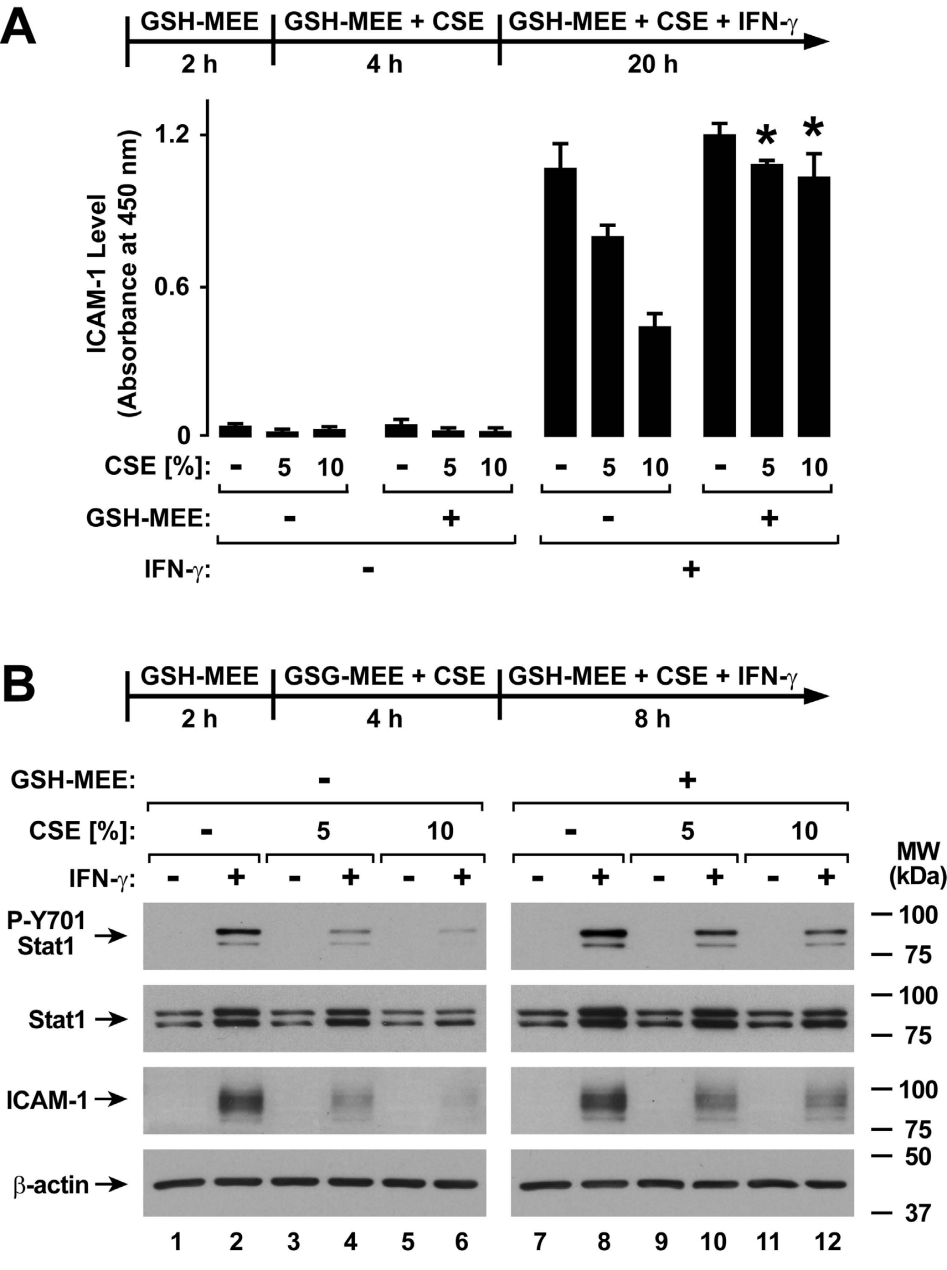
**GSH monoethyl ester inhibits cigarette smoke effects on type II interferon-induced responses**. *A*: ICAM-1 cell surface protein levels were determined using an enzyme-linked immunoassay with hTBE cells that were first treated in media without or with GSH-MEE for 2 hours. Cells were then incubated in media containing the antioxidant without or with CSE at the indicated concentrations for 4 hours, followed by incubation for 20 hours with the same compounds without or with IFN-γ. Values are expressed as mean ± S.D. (*n *= 3 replicates) and a significant difference (*p *< 0.01) in comparable CSE-treated levels between cells not incubated versus incubated with GSH-MEE is indicated by an *asterisk*. *B*: Tyrosine-701 phosphorylated and total Stat1, ICAM-1, and β-actin protein levels were assessed using immunoblot analysis of extracts from hTBE cells that were first treated in media without or with GSH-MEE for 2 hours. Cells were then incubated in media containing the antioxidant without or with CSE at the indicated concentrations for 4 hours, followed by incubation for 8 hours with the same compounds without or with IFN-γ.

**Figure 8:**
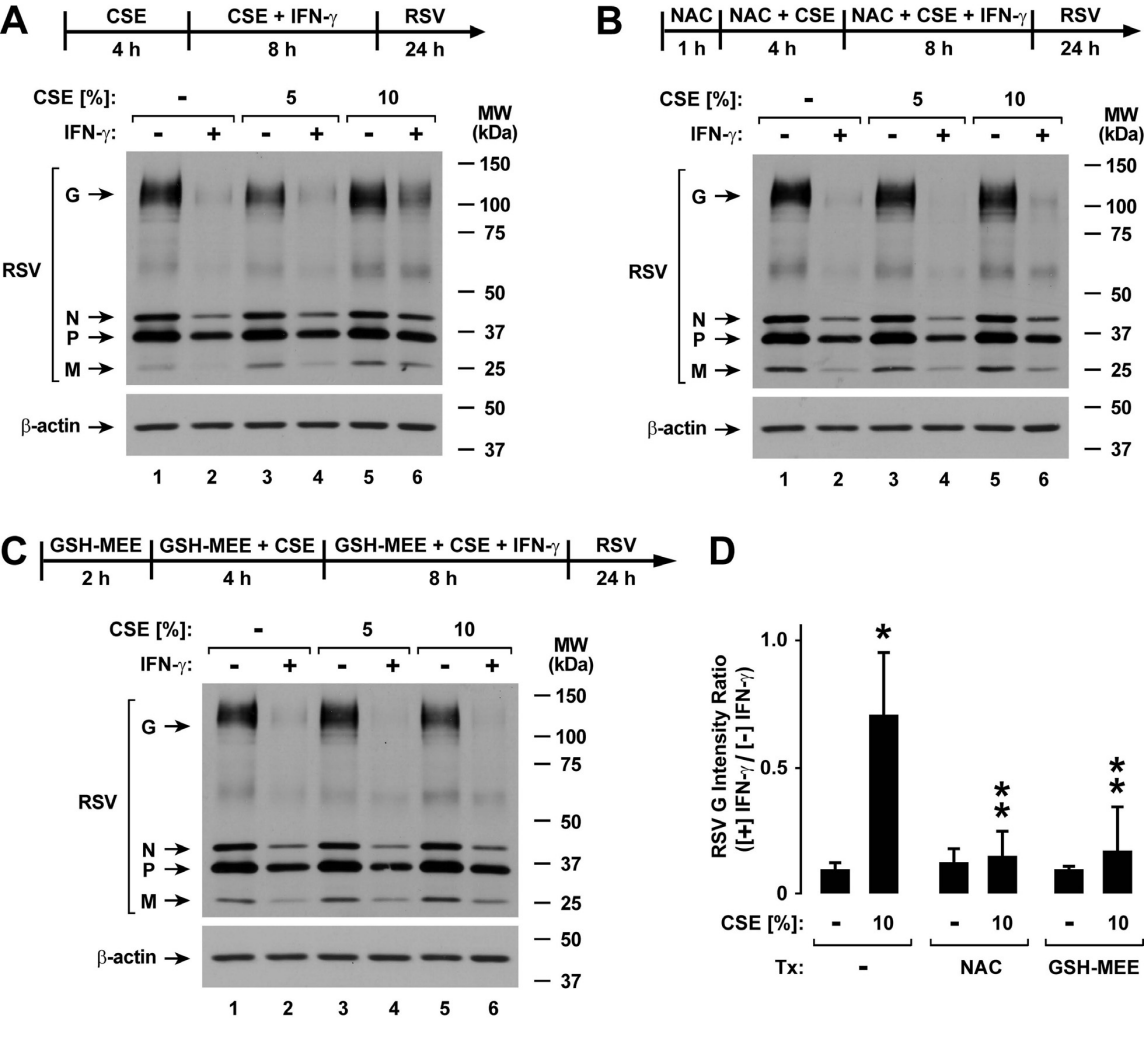
**N-acetylcysteine and GSH monoethyl ester inhibit cigarette smoke effects on antiviral defense**. RSV and β-actin protein levels were assessed using immunoblot analysis of extracts from hTBE cells that were first treated in media without (*A*) or with NAC for 1 hour (*B*) or GSH monoethyl ester for 2 hours (*C*). Cells were then incubated in media containing the same antioxidant without or with CSE at the indicated concentration for 4 hours, followed by incubation for 8 hours with the same compounds without or with IFN-γ. Cells were then infected with RSV for 24 hours.* D*: Protein levels were quantified using band densitometry of immunoblot analyses from separate experiments performed as outlined for *A*-*C*. The band intensities of RSV G/β-actin were compared between conditions by calculating ratios for each comparable condition in which cells were versus were not exposed to IFN-γ. Values are expressed as mean ± S.D. (*n *= 3 experiments), and a significant difference (*p *< 0.05) in levels between comparable antioxidant incubation conditions for cells without versus with CSE treatment is indicated by one *asterisk*, and in CSE-treated levels between cells not incubated versus incubated with NAC or GSH-MEE is indicated by two *asterisks*.

Cigarette smoke contains a variety of free radicals and highly reactive species that may affect cell function [31-33]. A pivotal system for cellular defense against oxidant stress is the glutathione antioxidant system [34]. Accordingly, we assessed the effects of glutathione supplementation using NAC or GSH-MEE on type II interferon-induced antiviral defense. Treatment of epithelial cells with NAC significantly decreased CSE effects on IFN-γ-induced ICAM-1 expression (Figure 6A). These results correlated with improved IFN-γ-induced Stat1 activation in NAC-treated hTBE cells exposed to either 5% or 10% CSE (Figure 6B). Similarly, GSH-MEE treatment of epithelial cells decreased the inhibitory effects of CSE on IFN-γ-induced ICAM-1 expression (Figure 7A) and Stat1 activation (Figure 7B). In order to assess the effects of these antioxidants on viral infection, NAC and GSH-MEE were tested using epithelial cells infected with RSV followed by viral protein detection using immunoblot analysis. In parallel experiments in which viral protein expression observed with IFN-γ pretreatment is decreased by CSE exposure (Figure 8A), treatment of hTBE cells with NAC (Figure 8B) or GSH-MEE (Figure 8C) restored high level IFN-γ inhibition of RSV protein expression in hTBE cells exposed to CSE (Figure 8D).

**Figure 9:**
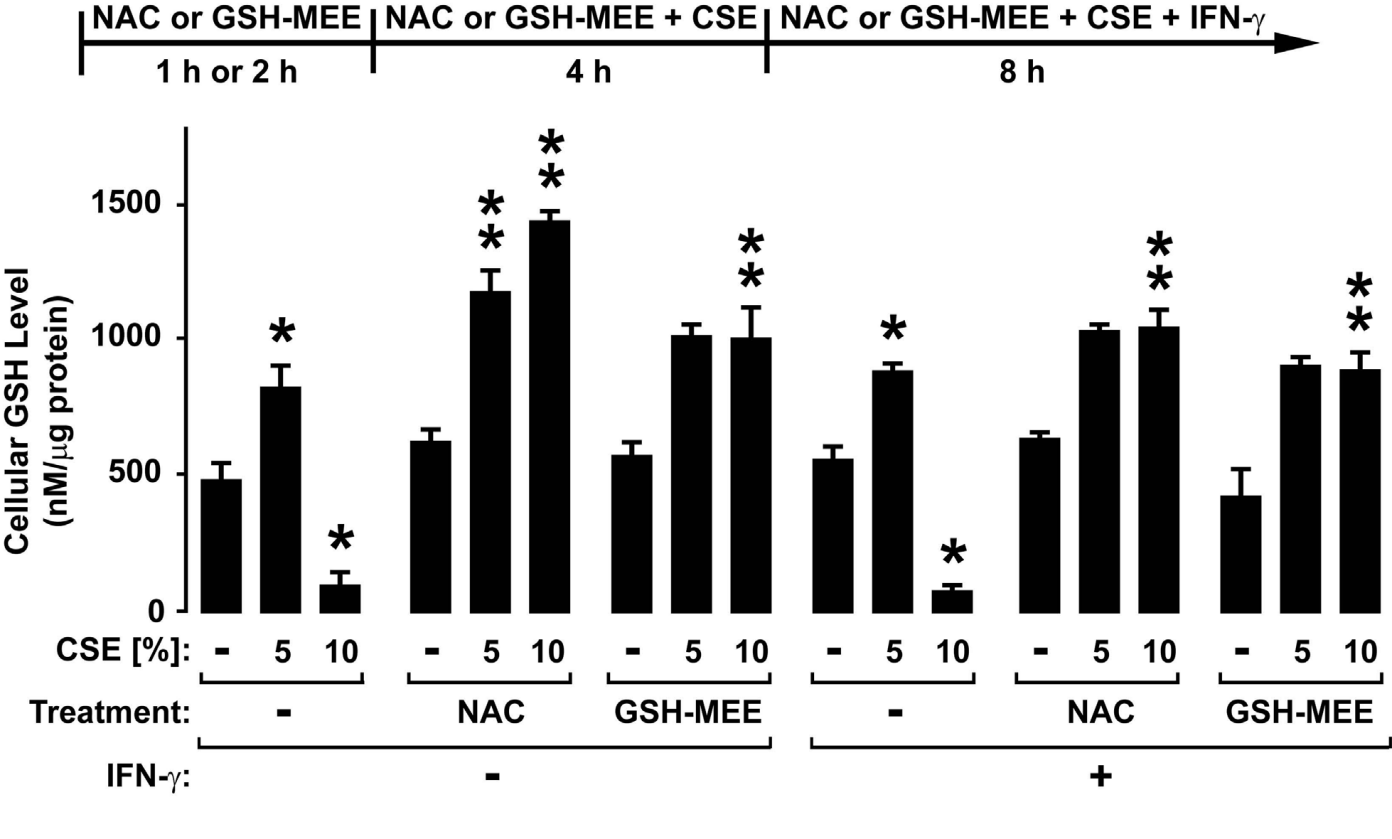
**Cigarette smoke extract alters epithelial cell glutathione levels**. Glutathione levels were determined using a luminescence-based assay with hTBE cells that were first treated in media without or with NAC for 1 hour or GSH-MEE for 2 hours. Cells were then incubated in media containing the antioxidant without or with CSE at the indicated concentration for 4 hours, followed by incubation for 8 hours with the same compounds without or with IFN-γ. Values are expressed as mean ± S.D. (*n *= 3 replicates), and a significant difference (*p *< 0.05) in uninduced or IFN-γ-induced levels between cells treated versus not treated with CSE but not exposed to antioxidant is indicated by one *asterisk*, and in comparable CSE-treated levels between cells not incubated versus incubated with NAC or GSH-MEE under uninduced or IFN-γ-induced conditions is indicated by two *asterisks*.

CSE, NAC, and GSE effects on glutathione levels in hTBE cells were also assessed. Interestingly, levels of the reduced form of glutathione in hTBE cells were increased by 5% CSE, but decreased by 10% CSE (Figure 9). Glutathione supplementation using NAC or GSH-MEE prevented the decrease in glutathione levels induced by 10% CSE treatment. Addition of IFN-γ had little effect under any of the conditions tested. These results indicate that antioxidants may be one strategy that could be used to inhibit effects of cigarette smoke on airway defense by restoring IFN-γ-dependent antiviral effects.

## Discussion

**Figure 10:**
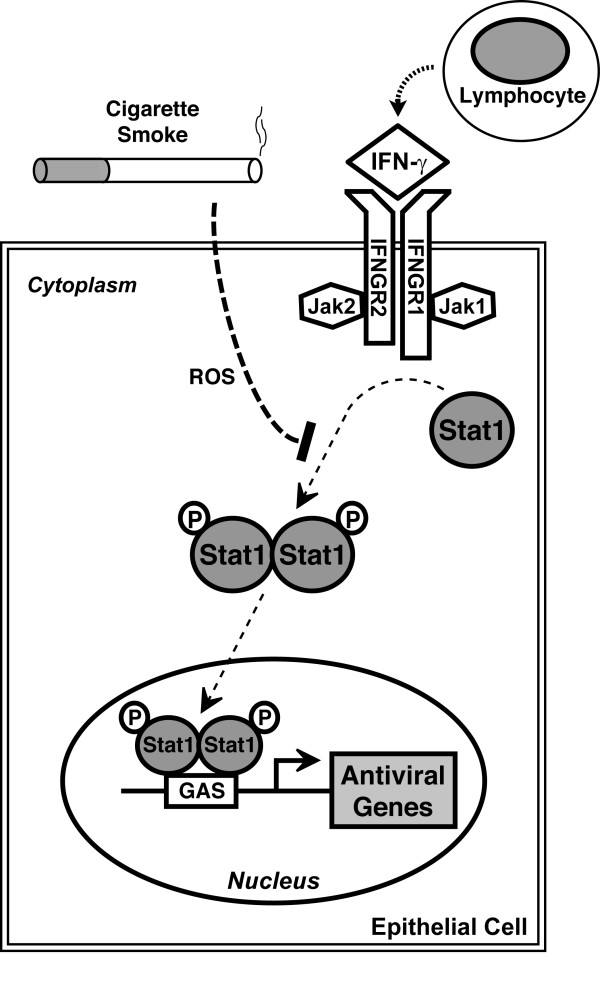
**Model for cigarette smoke effects on type II interferon signal transduction**. Cigarette smoke inhibits type II interferon-dependent gene expression by decreasing Stat1 phosphorylation. A portion of this effect is mediated by reactive oxygen species (ROS). Decreased antiviral gene expression decreases epithelial cell responses to IFN-γ that inhibit viral infection.

Epithelial cells in the airway are often targeted by respiratory viruses, and these cells actively participate in the antiviral response by responding to interferons and other mediators in the local environment, as well as responding directly to viral infection. Interferon-dependent immunity is critical for limiting and clearing viral infections, and it has been proposed that a prerequisite for successful viral invasion and replication in host cells is overcoming effects of interferons. Respiratory epithelium often has first contact and is the first line of defense against inhaled substances, and it is intuitive that cigarette smoke could directly affect epithelial cell functions when individuals smoke cigarettes. Our results indicate that CSE decreased the inhibitory effect of IFN-γ on epithelial cell infection by the respiratory pathogen RSV. CSE markedly inhibited IFN-γ-dependent Stat1 phosphorylation and gene expression, thereby providing a mechanism for CSE effects (Figure 10). CSE effects on IFN-γ-induced Stat1 activation, antiviral protein expression, and inhibition of RSV protein expression were decreased by glutathione augmentation, providing one strategy to alter cigarette smoke effects.

Cigarette smoke has been estimated to contain as many as 4,700 chemical compounds, including carbon monoxide, carbon dioxide, ammonia, methane, free radicals (e.g., superoxide and NO), and a variety of other highly reactive species such as aldehydes, semiquinones, and acrolein [31-33]. Cigarette smoke is conventionally described as having two phases: the tar phase (the > 1 μm particulate material trapped when smoke is passed through a standard Cambridge glass-fiber filter) and the gas phase (material that passes through the filter)[35]. The tar phase contains very high concentrations of radicals with the predominant species being the semiquinone radical, which is capable of reducing oxygen to superoxide and H_2_O_2_, and in the presence of free iron the highly reactive hydroxyl radical [32] The gas phase also contains various radical species, including NO and various carbon-based radicals, such as lipid peroxide radicals [36]. Some specific components of cigarette smoke have already been shown to affect antiviral defense function. For example, acrolein has been shown to suppress IFN-α-mediated antiviral defense against hepatitis C virus in human hepatocytes and enhance RSV replication in human airway epithelial cells [37,38]. Because cigarette smoke is a complex combination of many compounds that could affect epithelial cell functions in different ways, we felt it most valid to initially study the complete mixture in order to understand the overall effects of cigarette smoke on airway defense in humans.

Several models of cigarette smoke generation and cell exposure have been used in studies that assess biological effects. These vary from mixture of filtered or unfiltered cigarette smoke with media, solubilization of smoke material collected on a filter, direct cell exposure to cigarette smoke, as well as testing of individual components [37-41]. Each model has advantages and disadvantages that must be taken in to account when interpreting experimental results [42]. The system used for our studies utilized cigarette smoke exposure prior to and during interferon treatment based on the concept that epithelial cells in the airway are likely exposed to smoke prior to respiratory viral infection. We also tested cells exposed to CSE for 48 hours prior to treatments, reasoning that humans are often passively or actively exposed to cigarette smoke for longer durations. Epithelial cell exposure to CSE during viral infection was avoided because cigarette smoke can directly affect viral infection and replication [38].

Our results indicate that cigarette smoke effects on epithelial cell glutathione levels are concentration-dependent. Decreased glutathione levels that were observed with cell exposure to 10% CSE correlate with results in other reports, and likely are due to an increased oxidant-antioxidant ratio that overwhelms the ability of the glutathione system to detoxify CSE reactive species [43,44]. Conversely, many cigarette smokers have higher levels of GSH and this may correlate with our results using 5% CSE. Under these conditions, it is likely that low levels of cigarette smoke result in induction of the rate limiting enzyme in GSH synthesis, glutamate-cysteine ligase (formerly called γ-glutamylcysteine synthetase), through activation of the nuclear erythroid-related factor 2 (Nrf2) and AP-1 transcription factors [45,46]. These results indicate that cigarette smoke effects may not be completely due to reactive oxygen species as we saw some inhibition of interferon effects with 5% CSE even though there were increased cellular glutathione levels. Furthermore, treatments that increased cellular glutathione levels generally resulted in incomplete although significant restoration of IFN-γ effects. We also found that a prolonged CSE exposure duration with a period of epithelial cell exposure to both CSE and IFN-γ was required to inhibit IFN-γ-induced cell signaling. This characteristic likely explains the lack of CSE effects on type II interferon signaling reported previously [47]. This report also demonstrated that cigarette smoking condensate caused serine phosphorylation-dependent ubiquitination and degradation of the IFNAR1 subunit of the type I interferon receptor leading to attenuation of type I interferon antiviral responses in multiple cell lines. CSE requires time to activate an IFN-γ-modulating mechanism in order to affect this epithelial cell defense system, and we speculate that this is likely due to oxidant and nonoxidant-induced decreases in the level or activity of one or more type II interferon JAK-STAT signaling component (i.e., IFNGR1, IGNGR2, Jak1, and Jak2) or generation of a signaling inhibitor. Further studies are needed to determine the mechanisms for CSE effects on IFN-γ-induced Stat1 phosphorylation.

The results indicate that CSE directly inhibits antiviral effects of IFN-γ in epithelial cells through inhibition of the type II interferon JAK-STAT signaling cascade. However, other signaling pathways modulate IFN-γ-dependent responses, including p38 mitogen-activated protein (MAP) kinase, phosphatidylinositol 3 (PI3)-kinase, and protein kinase C isoforms [48-51]. Through alteration of these modulating pathways, CSE could indirectly affect IFN-γ-mediated immunity. Furthermore, there are multiple interferon pathways that control antiviral defense that could be affected by CSE. Recent reports indicate that type I interferon production, signal transduction, and antiviral effects are impaired in cells exposed to cigarette smoke condensate or conditioned media [47,52,53]. Altered interferon responses after cigarette smoke exposure may not be limited to epithelial cells. For example, macrophage and fibroblast cell lines exposed to cigarette smoke preparations in media and alveolar macrophages isolated from individuals that smoke cigarettes have reduced responsiveness to interferons [47,52,54-56]. Thus, cigarette smoke appears to affect multiple components of interferon-dependent antiviral defense. It is important to note that IFN-γ has other important functions in tissues besides antiviral defense. For example, IFN-γ is important for immune surveillance against malignant cells, and inhibition of interferon effects could be another mechanism through which cigarette smoke promotes lung carcinogenesis.

It appears that cigarette smoke has multiple effects that modify epithelial antiviral defense in the airway in addition to impairing interferon responses. Exposure to cigarette smoke leads to increased epithelial permeability both *in vivo *and *in vitro*, and this effect may amplify or mislocalize the airway defense response as well as allow viruses better access to their receptors on host cells [21,41,43,57]. Smoking is also known to alter clearance of microbes from the lungs via direct and indirect effects on mucociliary clearance [58]. Cigarette smoke has potent and widespread effects on both innate and adaptive immunity, including many effects on protease and inflammatory mediator levels released from epithelial cells *in vitro *and from lungs *in vivo *[39,59-61]. For example, cigarette smoke inhibits the production of some cytokines while promoting the production of others [62]. Specific components of cigarette smoke, such as NO_2_, ozone, and particulate matter, cause airway epithelial cell release of inflammatory mediators such as interleukin-8, granulocyte-macrophage colony-stimulating factor (GM-CSF), and tumor necrosis factor-α (TNF-α)[63,64]. Cigarette smoke increased inflammation in mice infected with high levels of influenza [60]. Furthermore, cigarette smoke has been shown to decrease pulmonary dendritic cells and antiviral immune responses in mice infected with adenoviral vectors [65]. Taken together, these and likely other undefined effects of cigarette smoke on defense function in airway epithelia lead to increased viral infection and/or altered inflammation. In support of this possibility, cigarette smoke extract has been shown to induce cellular effects that increase RSV replication and inflammatory mediator expression in epithelial cells [38,66]. Increased viral gene expression was also observed in neonatal mice exposed to cigarette smoke followed by infection with RSV [67]. The importance of each cigarette smoke effect on and antiviral immunity and inflammation likely differs in each individual based on many variables that include number of and manner that cigarettes are smoked, presence of underlying lung disease, and difference in antioxidant levels and defense mechanism competence among individuals.

## Conclusions

The results suggest that cigarette smoke alters type II interferon-dependent immunity in the airway resulting in increased incidence, duration, and/or severity or respiratory viral infections. A better understanding of mechanisms through which cigarette smoke impairs the host response to infection may allow for the development of therapeutic strategies that restore normal airway defense in individuals exposed to cigarette smoke.

## Competing interests

The authors declare that they have no competing interests.

## Authors' contributions

MAM did the glutathione analysis and a large portion of the signal transduction and protein analysis. LJM participated in study conception and data analysis, and performed cytotoxicity testing and a portion of the protein analysis including densitometry. SEM developed the assays of RSV infection and performed some of this analysis after CSE exposure. DCL conceived of the study, participated in its design and coordination, and wrote the manuscript. All authors read and approved the final manuscript.
